# Metagenomics and culturomics reveal the dual role of the gut microbiome in the development of immune-related toxicities and the efficacy of immune checkpoint inhibitors in cancer

**DOI:** 10.1186/s40168-026-02419-4

**Published:** 2026-05-04

**Authors:** Khoudia Diop, Myriam Benlaïfaoui, Sébastien Hunter, Eder Orlando Méndez-Salazar, Taiki Hakozaki, Corentin Richard, Diogjena Katerina Prifti, Sylva Kourtian, Francis Proulx-Rocray, Sabrine Naimi, Mayra Ponce, Meriem Messaoudene, Florent Cauchois, Wiam Belkaid, Veronique Bataille, Karla Lee, Catalin Mihalcioiu, Ian Robert Watson, Arielle Elkrief, Bertrand Routy

**Affiliations:** 1 University of Montreal Hospital Research Centre (CRCHUM), Montreal, Quebec Canada; 2 Hematology-Oncology Division, Department of Medicine, University of Montreal Hospital Centre (CHUM), Montreal, Quebec Canada; 3 Department of Twin Research and Genetic Epidemiology, King’s College London, London, United Kingdom; 4 Research Institute of the McGill University Health Centre, Montréal, Quebec Canada; 5 Rosalind and Morris Goodman Cancer Institute, McGill University, Montréal, Quebec Canada

**Keywords:** Gut microbiome, Cancer, ICI, IrAE, Colitis, Culturomics, Metagenomics, Melanoma, NSCLC

## Abstract

**Background:**

Despite their major impact on cancer treatment, immune checkpoint inhibitors (ICI) are frequently associated with immune-related adverse events (irAE). Growing evidence suggests that the occurrence of irAE may be correlated with enhanced ICI efficacy, although the underlying mechanisms remain unknown. Most studies investigating the role of the gut microbiome in oncology have relied on sequencing approaches, particularly shotgun metagenomics. Although microbiome profiling revealed strong associations between specific bacterial taxa and clinical outcomes, it has limitations, including an inability to detect low-abundance bacteria and to recover live cultivable bacteria. To overcome these limitations, we combined shotgun metagenomics and culturomics on fecal samples collected from patients with melanoma and non-small cell lung cancer (NSCLC), at baseline and at the onset of immune related (ir)-colitis.

**Results:**

We first validated across three independent cohorts of 589 patients with melanoma or NSCLC treated with ICI that grade ≥ 2 irAE were associated with significantly longer overall survival (OS) and progression-free survival (PFS). Complementary analysis using shotgun metagenomics and culturomics revealed that patients who developed grade ≥ 2 irAE had a lower alpha diversity compared to those who did not develop grade ≥ 2 irAE. Metagenomics results showed enrichment of *Ruminococcus gnavus* and *Streptococcus vestibularis* at baseline in grade ≥ 2 irAE patients, while *Clostridium paraputrificum* and *Streptococcus* spp. were isolated by culturomics from baseline stool samples from ir-colitis patients. Longitudinal analysis of paired stool samples revealed a shift in microbiome composition with enrichment of *Paraclostridium bifermentans* and *Clostridium paraputrificum*, lower lipopolysaccharide and higher flagellin concentrations at baseline compared with the time of ir-colitis. Fecal microbiome transplantation from a patient with ir-colitis into mice induced surrogate markers of colonic inflammation and enhanced the anti-tumor activity of combined anti-PD-1/CTLA-4. *P. bifermentans* isolated from this patient sample demonstrated direct epithelial barrier disruption in Caco-2 monolayers, characterized by decreased ZO-1 and Occludin immunofluorescence signal and increased *TNF-α* and *IL-1β* expression. Moreover, in the dextran sodium sulfate (DSS) colitis model, *P. bifermentans* worsened weight loss. In a separate tumor model, it amplified the anti-tumor effect of dual ICI. This beneficial effect was also maintained after treatment with *P. bifermentans* < 3 kDa filtered supernatant.

**Conclusion:**

Altogether, our results suggest that *P. bifermentans* promotes subclinical colitis while increasing the efficacy of dual ICI. This provides a potential microbiome-derived link between irAE and improved anti-tumor responses.

Video Abstract

**Supplementary Information:**

The online version contains supplementary material available at 10.1186/s40168-026-02419-4.

## Background

Immune checkpoint inhibitors (ICI) have transformed the treatment landscape for patients with melanoma and non-small cell lung cancer (NSCLC), leading to durable survival benefits in both advanced and early-stage disease [1, 2]. However, ICI can also trigger immune-related adverse events (irAE), inflammatory toxicities that affect normal tissues due to immune activation in the absence of self-tolerance checkpoints [3]. These off-target toxicities most commonly affect barrier tissues such as the skin and gastrointestinal tract, with immune-related diarrhea or colitis (ir-colitis) observed in 30–40% of cases, particularly in patients receiving anti-CTLA-4 therapy [4]. Clinically meaningful irAE, classified as grade 2 or higher according to the Common Terminology Criteria for Adverse Events (CTCAE), occur in 20% to 60% of patients, depending on whether they are receiving combination ICI therapy [5]. Management of severe irAE typically requires immunosuppression with corticosteroids [6]. In cases refractory to steroids, escalation to second-line immunosuppressive agents, such as infliximab (a monoclonal antibody targeting TNF-α) or vedolizumab (an anti-integrin α4β7 antibody with gut-specific activity), is recommended [7].

Beyond toxicity, recent studies have linked the gut microbiome to response to ICI, with beneficial bacteria enriched in responders, while immunosuppressive bacteria were more prevalent in non-responders, highlighting their potential role as predictive markers of clinical benefit [8, 9]. These findings were recently translated into a qPCR-based assay to rapidly assess microbiome composition for predicting patient responses to ICI and are currently being evaluated as a point-of-care test in a trial [9]. As a result, several microbiome-targeted strategies, including fecal microbiota transplantation (FMT), dietary intervention, and probiotics, are being developed as potential therapeutic adjuvants to ICI [10–13].

Given the key role of the gut microbiome in shaping anti-tumor immune responses, it is not surprising that microbial composition has also been implicated in the development of irAE. Interestingly, the fact that irAE frequently affect tissues with mucosal or environmental interfaces suggests that external factors may contribute to their development. Among these, the gut microbiome has emerged as a compelling candidate. For example, in melanoma patients, loss of *Faecalibacterium prausnitzii* and enrichment of *Bacteroides intestinalis* and *Bacteroides clarus* were associated with irAE development [14–16]. In preclinical murine models of RET-driven tumors, *B. intestinalis* was implicated in the development of subclinical colitis after anti-PD-1 and anti-CTLA-4 therapy [14]. Other bacteria, including *Streptococcus* spp. and *Lachnospiraceae* spp., have also been associated with the development of irAE in melanoma patients treated with anti-PD-1 monotherapy or combined with anti-CTLA-4 [16]. Moreover, Lo et al. in the context of anti-PD-1/CTLA-4 blockade, confirmed that mice colonized with wild-derived microbiome developed a more severe ir-colitis, compared to standard laboratory mice, through the activation of IFN-γ producing CD4^+^ Th1 cells and reduction of gut microbiome-dependent RORγt^+^ T regulatory cells (Treg) [17]. The role of Treg in microbiome-dependent protection of ir-colitis was previously observed by Wang et al. demonstrating a positive association between *Bifidobacterium* abundance and Treg expansion in mice in the context of CTLA-4 blockade [18]. Mechanistically, Sun et al. demonstrated that *Bifidobacterium* administration to dextran sulfate sodium (DSS) mice model treated with anti-CTLA-4 can alter the gut microbiome composition by increasing *Lactobacillus* and other probiotics abundance in a Treg-dependent manner, while activating the IL-10/IL-10R suppressive pathway in Treg, thereby enhancing control of inflammatory ir-colitis [19].

In addition to their link with the gut microbiome, the occurrence of irAE has been associated with improved clinical outcomes [20]. Several meta-analyses from cancer cohorts across distinct malignancies and geographies have demonstrated that patients experiencing irAE exhibit longer progression-free survival (PFS) and overall survival (OS), suggesting a positive link between irAE and therapeutic efficacy [21, 22]. However, the strength of this association is debated, especially when the immortal time bias is taken into consideration, whereby patients who rapidly progressed and died before irAE onset are excluded from the irAE group, leading to an overestimation of the apparent survival benefit [23–25]. This association between toxicity and efficacy has raised important questions about whether overlapping immune mechanisms drive both therapeutic responses and irAE. Moreover, FMT has shown early promising potential in treating patients with steroid-refractory ir-colitis, reinforcing the relevance of the gut microbiome in both ICI efficacy and irAE biology [10, 26–28].

In this study, we sought to dissect the relationship between the gut microbiome, irAE, and ICI outcomes using a combined shotgun metagenomics and culturomics-based stool profiling from the samples of patients with cancer. Our data indicate that patients who developed irAE exhibited reduced baseline microbial diversity yet achieved improved clinical outcomes following ICI therapy. Through culturomics, we isolated *Paraclostridium bifermentans* from the stool of patients with ir-colitis and demonstrated that this bacterium exacerbated inflammation in vitro and in DSS murine models while also enhancing anti-tumor responses to ICI. These findings underscore the dual role of specific gut bacteria in modulating ICI efficacy and irAE pathogenesis, offering a microbiome-derived mechanism underlying treatment response and toxicity.

## Material and methods

### Human cohorts

#### Patient recruitment

Patients with advanced melanoma or non-small cell lung cancer (NSCLC) receiving immune checkpoint inhibitors were recruited from the Montreal University Hospital Center, McGill University and King’s College London. Baseline clinical characteristics were recorded, and stool samples were collected before and after the start of treatment. The study was approved by the institutional ethics committee (CHUM Research Center: MP-02–2021-9334, McGill: 2019-4778, King’s College London: NCT03643289) and conducted in accordance with the Declaration of Helsinki and the guidelines of Good Clinical Practice. All patients provided written, informed, and signed consent prior to study participation.

#### Toxicity grading and clinical outcomes

Patients were monitored for irAE during treatment, and toxicity grading was performed according to CTCAE v5.0. Clinical responses were monitored using the Response Evaluation Criteria in Solid Tumors (RECIST) v1.1 through radiological assessments at predefined intervals.

Because irAE typically occur early after ICI initiation, and because patients with very poor prognosis may die before having the opportunity to develop an irAE, Kaplan–Meier survival comparisons between irAE groups are susceptible to immortal time bias. To reduce this bias, we excluded patients with an overall survival (OS) inferior to 3 months from Kaplan–Meier analyses, regardless of irAE status, consistent with prior analyses using a similar approach [24, 25].

#### Fecal metagenomics profiling

Baseline stool samples were collected, initially stored at 4 °C and then kept at − 80 °C. Microbial composition in fecal samples was assessed using shotgun metagenomics sequencing. DNA extraction was performed using DNeasy PowerSoil Pro Kit (Qiagen) following the manufacturer’s instructions. Sequencing libraries were prepared using Illumina® DNA Prep (M) Tagmentation kit (Illumina), with dual-indexed adapters for multiplexing. Samples were sequenced using NovaSeq 6000 S4 platform (Illumina), generating 150 bp end-paired reads. Raw sequencing data underwent quality control and preprocessing using the Segata Lab pipeline (https://github.com/SegataLab/preprocessing), which included the removal of low-quality reads, adapters, and potential contaminants. To eliminate host-derived sequences, reads were aligned against the human genome (hg19) and phiX174 spike-in controls using Bowtie2 (v2.3.4.1), and matching sequences were removed. Microbial taxonomic profiling and quantification were conducted using MetaPhlAn 4, providing absolute read counts and relative abundances for each sample.

#### Fecal microbial culturomics process

In addition to metagenomics, the isolation and characterization of intestinal bacteria were carried out using the microbial culture approach, as described previously [29]. Five culture conditions were used: direct culture, 28 °C and 37 °C culture bottles with defibrinated sheep blood and rumen under aerobic and anaerobic atmospheres. All isolated bacterial colonies were identified using Matrix-Assisted Laser Desorption/Ionization Time-of-Flight mass spectrometry and/or 16S rRNA gene sequencing. Paired samples (baseline and acute colitis) were analyzed simultaneously using the same procedure to avoid bias between groups. The pure culture of all isolates was stored in − 80 °C as a glycerol stock for downstream experiments.

#### Fecal lipopolysaccharide (LPS) and flagellin levels

Fecal LPS and flagellin levels were quantified using human embryonic kidney (HEK)-Blue-mTLR4 and HEK-Blue mTLR5 cells, respectively (Invivogen, San Diego, CA, USA) according to the manufacturer's instructions. Stool samples from the same patient at baseline and during acute colitis were resuspended in PBS to a final concentration of 100 mg/mL and vortexed briefly for 10 s without adding beads to avoid bacterial disruption [30]. The homogenate was then centrifuged for 2 min at 8000×*g*. Serial dilutions of the resulting supernatant were applied to HEK-Blue-mTLR4 and HEK-Blue mTLR5 cells. Purified Escherichia coli LPS and flagellin (Sigma-Aldrich) were used for standard curve determination, respectively. After 24 h of stimulation, the cell culture supernatant was applied to QUANTI-Blue medium (Invivogen), and alkaline phosphatase activity was measured at 620 nm after 30 min.

### In vivo murine studies

#### Mice and housing conditions

All animal studies were approved by the *Comité Institutionnel de Protection des Animaux* (CIPA, ethics number: C22052BRs) and conducted in compliance with Canadian laws and regulations. Wild-type female C57BL/6 mice, 6 weeks old, were purchased from Charles River and housed in specific pathogen-free (SPF) and ventilated cages with free access to water and a regular chow diet. After 2 days of acclimatization, they were randomly divided into groups of 5 animals per cage. Every 2–3 days, the mice were weighed, and their fecal samples were also collected for 16S rRNA gene sequencing or lipocalin analysis. All mouse experiments were performed in the CRCHUM animal facility under standard conditions: 12 h light/dark cycle, room temperature of 25 °C and humidity between 40 and 60%.

#### Fecal microbiota transfer (FMT)

Mice were first treated with an antibiotic (ATB) cocktail (1 mg/mL ampicillin, 1 mg/mL colistin, and 5 mg/mL streptomycin; Sigma-Aldrich) in their drinking water for 3 days. ATB efficacy was confirmed by seeding mice feces on Columbia agar followed by 48 h incubation under anaerobic and aerobic conditions as previously described [31]. Next, fecal material from colitis patients was suspended in sterile 0.9% saline, mixed well, and allowed to settle to remove large particles. Two hundred microliters of the fecal suspension were administered by oral gavage to recipient mice, and 100 μL were applied to their fur. Control mice received an equivalent volume of sterile saline solution after the ATB. Two weeks after FMT, tumor cells were injected into the mice.

#### Cell line, subcutaneous tumor injection, and ICI treatments

The murine fibrosarcoma cell line MCA-205 was cultured in Roswell Park Memorial Institute (RPMI; Gibco) 1640 medium supplemented with 10% fetal bovine serum (FBS), 1% penicillin/streptomycin, and 1% L-glutamine. Cultures were maintained at 37 °C in a 5% CO2 atmosphere. Mice were subcutaneously inoculated in the right flank with 0.8 × 10^6 MCA-205 cells. When the tumors' surface reached 30 to 35 mm^2^, mice were treated intraperitoneally with an anti-PD-1 antibody (250 μg/mouse; clone RMP1-14) in combination with an anti-CTLA-4 antibody (100 μg/mouse; clone 9D9). Isotype control groups were treated with isotype PD-1 (clone 2A3) and isotype CTLA-4. Mice received four injections of the treatments (anti-PD-1/CTLA-4 or isotype control) at 3-day intervals. On each treatment day, feces were collected, and tumor size was measured using a caliper. All antibodies were purchased from BioXcell, NH, USA.

#### DSS-induced colitis and determination of colitis incidence

Mice received 2–2.5% dextran sodium sulfate (DSS, MP Biomedicals, USA) in drinking water for 7 days. After this period, DSS-containing water was replaced with regular drinking water for the remainder of the experiment. Body weight, stool consistency, and the presence of blood were monitored for each mouse every 3 days from the start of the experiment until the end.

#### Bacteria culture and preparation

*Paraclostridium bifermentans* strain kd544 and *Clostridium paraputrificum* strain kd463 were isolated from patient stool samples during the culturomics analysis. These bacteria were cultured anaerobically on a Columbia agar plate for 24 h at 37 °C in an anaerobic chamber (Bactron900) containing a gas mixture of 10% CO_2_, 10% H2, and 80% N2. On the day of oral gavage, pure colonies of each bacterium were suspended in sterile 0.9% saline, and the OD_600_ was stabilized at 0.4.

#### Cell-free supernatant and heat-killed *P. bifermentans* preparation

*P. bifermentans* was grown in brain heart infusion (BHI) at 37 °C under anaerobic conditions for 24 h. When the culture reached an OD_600_ of 0.4, the broth was centrifuged at 2500 rpm, 4 °C for 15 min. To prepare heat-killed *P. bifermentans*, after centrifugation, the pellet was washed 2 times with PBS before being suspended in BHI and heated at 95 °C for 30 min. For cell-free supernatant (CFS), the collected supernatant was filtered through a 0.2-μm pore filter to remove all traces of microorganisms. The fraction below 3 kDa (< 3 kDa) was obtained after refiltration of *P. bifermentans* CFS using 3 K protein concentrator columns (ThermoScientific).

#### Bacteria oral gavage procedure

Mice were treated with 100 µL of *P. bifermentans* or *C. paraputrificum*, heat-killed *P. bifermentans* or CFS *P. bifermentans* by oral gavage. The gavage was performed every other day. Control groups received an equivalent volume of sterile 0.9% saline or BHI, depending on the experiment.

#### 16S rRNA gene sequencing and data analysis

Mice fecal samples were collected and stored at − 80 °C until use. Genomic DNA (gDNA) extraction was performed using the Quick-DNA Fecal/Soil Microbe Microprep Kit according to the manufacturer’s instructions (Zymo Research, CA, USA). gDNA was quantified using a NanoDrop™ spectrophotometer (Thermo Fisher Scientific). To characterize the gut microbiome composition, 16S rRNA gene sequencing targeting the V3–V4 region was performed via *Génome Québec* core service. Briefly, the V3–V4 region of the 16S rRNA gene was amplified from the extracted and purified gDNA using the 341 F and 805R primer pairs, followed by sequencing on an Illumina MiSeq platform (Illumina, CA, USA). Paired-end reads were denoised, filtered, trimmed, and merged using the DADA2 package (v1.17) in R. Taxonomic assignment was performed using the RDP classifier (v2.2) trained on the SILVA database (v138). Observed, Shannon, and Inverse Simpson indices were calculated to assess alpha diversity. Beta diversity was assessed using Bray–Curtis dissimilarity and visualized by principal coordinates analysis (PCoA). Differentially abundant taxa between groups were identified using LEfSe.

#### Fecal lipocalin 2 (Lcn-2) quantification

Mice fecal samples were reconstituted in PBS containing 0.1% Tween-20 to a final concentration of 100 mg/mL and vortexed at 37 °C for 30 min to get a homogenous fecal suspension. The homogenate was then centrifuged for 10 min at 4 °C and 12,000 rpm. Clear supernatants were collected, and Lcn-2 levels were quantified in these supernatants using Duoset murine Lcn-2 ELISA kit (R&D Systems, Minneapolis, USA).

### In vitro experiments

#### Caco-2 cells differentiation

Caco-2 colonic tumor cell lines were cultured in T75 cm^2^ culture flasks (Corning, NY, USA), in high-glucose Dulbecco’s modified Eagle’s medium (DMEM, Corning) supplemented with 10% heat-inactivated fetal bovine serum (FBS, Gibco, NY, USA), 1% L-glutamine (Gibco, NY, USA), 1% of MEM NEAA (Gibco, NY, USA) and 1% antibiotic cocktail (100 μg/mL streptomycin and 100 μg/mL penicillin), referred to as complete medium. Cultures were maintained at 37 ˚C in a humidified atmosphere (95% air + 5% CO_2_).

For differentiated cells, 6-well plates or cover slips were coated with 50 μg/mL of Type I Collagen (Corning®, Rat tail) diluted in sterile PBS and incubated for 1 h at room temperature. After coating, the collagen solution was removed, and the 6-well plates or the coverslips were washed before 200,000 of Caco-2 cells were seeded onto them. Medium was changed every 2 days, and cells were passaged when they reached a maximum density of about 80%. Differentiation was completed 19 days post-confluency.

#### Caco-2 and bacteria co-culture

After completion of Caco-2 differentiation, coverslips containing confluent differentiated cells were co-cultured with freshly prepared bacterial suspensions adjusted to an OD_600_ of 0.4 for 4 h. Following incubation, coverslips were washed twice with PBS, fixed, and processed for immunofluorescence analysis. For RNA extraction experiments, cells were detached from the coverslips by trypsinization, collected, and centrifuged. The resulting cell pellets were stored at − 80 °C until further processing.

#### RNA extraction and RT-qPCR

Total RNA was extracted from Caco-2 cells using the RNAeasy kit (Qiagen) according to the manufacturer’s instructions. Complementary DNA (cDNA) was synthesized from 1 µg of total RNA using SuperScript VILO cDNA kit (Thermo Fisher Scientific). Quantitative PCR was then performed using qPCRBIO SyGreen Blue Mix Hi-ROX (PCRBIOSYSTEMS) on a CFX384 Touch Real-Time PCR Detection System (Biorad). Gene expression of *IL-1β* (forward: 5′-CCAGGGACAGGATATGGAGCA-3′; reverse: 5′-TTCAACACGCAGGACAGGTACAG-3′) and *TNF-α* (forward: 5′-CTGCCTGCTGCACTTTGGAG-3′; reverse: 5′-ACATGGGCTACAGGCTTGTCACT-3′) was normalized to the reference genes *ACTB* (forward: 5′-CTGGAACGGTGAAGGTGACA-3′; reverse: 5′-AAGGGACTTCCTGTAACAATGCA-3′) and *HPRT1* (forward: 5′-AGATGGTCAAGGTCGCAAG-3′; reverse: 5′-GTATTCATTATAGTCAAGGGCATATCC-3′). Relative gene expression was calculated using the 2^-ΔΔCt method and expressed as fold change relative to the control condition.

### Immunofluorescence

Fixation of cells was performed using 4% paraformaldehyde, followed by permeabilization with 0.1% Triton X-100 for 3 min. Post-fixation, coverslips were blocked with 3% bovine serum albumin (BSA) in PBS containing 0.2% Tween-20 for at least 30 min before being incubated overnight at 4 °C with the following conjugated antibodies: ZO-1 (ZO1-1A12, Thermo Fisher Scientific) and Occludin (OC-3F10, Thermo Fisher Scientific). Antibodies were used at a concentration of 2 μg/mL unless specified otherwise. Chromatin was stained with Hoechst 33,342 (Sigma-Aldrich).

### Confocal microscopy

Imaging was performed using either an inverted spinning-disk confocal microscope Zeiss Axio Observer.Z1 equipped with a Yokogawa CSU-X1 spinning disk confocal module (Zeiss, Germany) and a motorized stage (ASI system with an MCL nano-drive piezo for precise focus control) or a laser scanning inverted confocal microscope Leica Stellaris 8 DMI8-CS (Leica Microsystem). For spinning disk confocal imaging, images were acquired using ZEN 2.6 (Blue Edition) software (version 2.6.76.00000). All channels were acquired sequentially. For Alexa Fluor 647 and Alexa Fluor 488, excitations were provided by diode lasers at 639 nm and 488 nm, respectively, controlled by acousto-optic tunable filters (AOTF). Emission was collected using a dual band pass filter (DBP 527/54 + 645/60 from Chroma). For DAPI, excitation was provided by diode lasers at 405 nm, controlled by acousto-optic tunable filters (AOTF). Emission was collected using a dual band pass filter (DBP 460/30 + 590/30 from Chroma). Detection was performed using an EMCCD Evolve 512 monochrome camera (Photometrics) with a native pixel size of 16 µm, a 512 × 512 resolution, 16-bit depth, and a 1.2 × EMCCD camera adapter. A Fluar 40 ×/1.3 Oil M27 objective (Zeiss, Germany) was used, with a physical pixel size of 0.333 × 0.333 µm in the XY plane and a 1.20/0.93/0.84 µm depth of focus for AF647/AF488/DAPI, respectively. Four Z-stacks were acquired to produce a full orthogonal projection of the entire layer of cells. For Stellaris 8 imaging, images were acquired using LasX software (Version 4.6.1.27508) and a HC PL-Apo CS2 63x/1.40 Oil objective. For Alexa Fluor 488, excitation was provided by an OPSL 488 nm laser in a first sequence, and in a second sequence for DAPI and Alexa Fluor 647, excitation was provided by diode lasers at 405 nm and 639 nm, respectively. All lasers were controlled by acousto-optic tunable filters (AOTF). A double dichroic mirror 488/638 was used in both sequences. All detections were collected using spectral HyDS detectors (Leica Microsystem). For AF488, emission was collected between 493 and 559 nm, for DAPI between 415–559 nm and for AF647 between 646 and 844 nm. Images were acquired at 12 bits, 2096 × 2096 xy format (60 nm pixel size) with a 1.5 zoom and at 400 Hz scan speed in a bidirectional mode. A line average × 6 was applied for all sequences. Z-stack were acquired with a fixed z-step of 2.6 µm to acquire the entire thickness of cell layers.

### Image quantification

Unless specified otherwise, all experiments and statistical evaluations were carried out in triplicate with independent biological samples. Image processing was performed using ImageJ2, and all images within a given figure were scaled consistently. Post-acquisition analysis was conducted with ImageJ2 (Fiji version 1.53c). Brightness and contrast adjustments were applied uniformly across datasets to enhance image clarity while preserving data integrity. Greyscale images were combined into z-stack projections using the maximum intensity method to enhance signal clarity from the cell membrane regions. For each condition, 5 stacks were analyzed, focusing on 5 different areas per condition. The data are expressed as the fluorescence intensity in arbitrary units.

### Statistical analysis

#### Clinical and experimental data analyses

Clinical and experimental data were analyzed using GraphPad Prism (v10.5.0, GraphPad Software, San Diego, CA, USA). Continuous variables were compared using Student’s *t*-test or one-way ANOVA for normally distributed data, and Mann–Whitney *U* or Kruskal–Wallis tests for non-parametric data. Survival curves were generated with the Kaplan–Meier method and compared using the log-rank test. Data are presented as mean ± SEM, and *p* < 0.05 was considered statistically significant; *p* > 0.05 was considered non-significant (ns).

#### Metagenomics analyses

All microbiome statistical analyses and visualizations were performed in R (4.4.2). Sequencing data were processed with phyloseq, with taxonomic assignments and abundance tables imported into a phyloseq object including sample metadata. Alpha-diversity indices (richness, Shannon) were computed using the vegan package, and differences between groups were tested with non-parametric tests (*p* < 0.05 considered significant). Beta-diversity was assessed using Bray–Curtis dissimilarity and visualized with Principal Coordinates Analysis (PCoA); group differences were evaluated with PERMANOVA (adonis function, 999 permutations). Differential abundance analysis was conducted using LEfSe (package yingtools2), retaining features with LDA score > 2 and *p* < 0.05. Multivariable modeling was performed using MaAsLin2, with the irAE/response group as reference when indicated. Taxonomic abundance patterns were visualized by heatmaps generated with ComplexHeatmap, based on taxa differentially abundant between conditions (Wilcoxon rank-sum test, *p* < 0.05).

#### Culturomics analyses

Alpha and beta diversities, as well as group-specific prevalence species, were assessed to visualize differences in gut microbiome after culturomics. Alpha diversity was calculated as species richness, defined by the presence/absence of unique bacterial species identified by MALDI-TOF MS in each sample. Species richness was compared between groups (No ir-colitis vs ir-colitis; baseline ir-colitis vs acute ir-colitis). Statistical analysis was chosen according to data distribution, using the Shapiro–Wilk test on GraphPad Prism version 10. For normally distributed data, a Student’s *t*-test was used. On the other hand, for non-normally distributed data, the non-parametric Mann–Whitney test was used. Beta diversity was calculated using presence/absence-based Jaccard distances and visualized with principal coordinates analysis (PCoA) ordination using R software version 4.4.0. The difference in microbial composition between groups was quantified with Permutational Analysis of Variance (PERMANOVA) using the adonis2 function of the vegan package. Based on their absence and presence, the prevalence of each species in each group was determined, and the difference was calculated to identify the enriched taxa in each group. Statistical significance of prevalence differences was assessed using the chi-squared test with a false discovery rate (FDR) adjustment using the method of Benjamini and Hochberg. In addition, a Venn diagram was used to view specific and common species between groups.

## Results

### Development of irAE is associated with improved clinical outcomes but reduced microbiome diversity

**Fig. 1:**
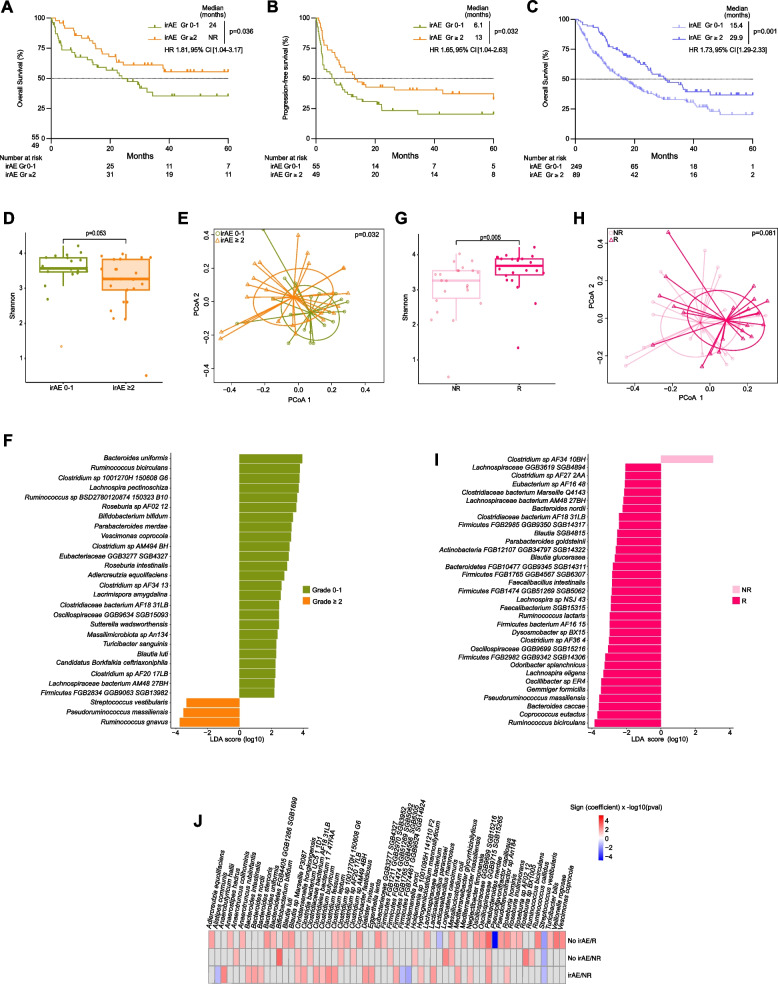
Development of irAE is associated with improved clinical outcomes but reduced microbiome diversity. **A** Overall survival and **B** Progression-free survival according to the occurrence of irAE grade 0–1 (*n* = 55) and grade ≥ 2 (*n* = 49). Statistical differences were assessed using a log-rank test (*p* < 0.05 considered significant). **C** Overall survival of Canadian NSCLC patients stratified by irAE grade 0–1 (*n* = 249) vs grade ≥ 2 (*n* = 89). Shotgun metagenomic analysis showing **D** alpha diversity using Shannon index comparison between patient groups, and **E** beta diversity assessed using principal coordinates analysis (PCoA) based on Bray–Curtis dissimilarity, and ellipses indicate the 95% confidence interval of group centroids. Each point represents one fecal sample. **F** Linear discriminant analysis (LDA) effect size (LEfSe) plot showing the differentially abundant bacterial taxa after stratification of patients into Grade 0–1 (*n* = 20) and Grade ≥ 2 (*n* = 25) groups. The *x*-axis represents the LDA score (log 10), indicating the magnitude of the difference in relative abundance. **G** Alpha diversity, **H** Beta diversity (PCoA Bray–Curtis), and **I** LEfSe representation of microbiome composition of the advanced melanoma patients segregated based on median PFS. R: patients above the PFS median (*n* = 22) and NR: below the median PFS (*n* = 22). **J** Heatmap representation of bacterial species identified by MaAsLin multivariable modeling, comparing patients with no irAE and response (No irAE/R), no irAE and no response (No irAE/NR), and irAE without response (irAE/NR). Colors represent signed statistical significance, calculated as sign(coefficient) × − log10(*p*-value) from MaAsLin models, relative to the reference group (irAE/R). Positive values (red) indicate taxa enriched in the indicated group, whereas negative values (blue) indicate taxa depleted relative to irAE/R

To validate the association between irAE and response to ICI, we retrospectively collected clinical data from 104 patients treated in Canada with advanced melanoma amenable to ICI. Among them, 29 patients (28%) received combined ipilimumab (anti-CTLA-4) and nivolumab (anti-PD-1) treatment. Baseline characteristics are presented in Table S1. In total, 49 patients (47%) experienced grade ≥ 2 irAE. Patients who experienced grade ≥ 2 irAE had significantly improved clinical outcomes compared to those without such events. Median overall survival (OS) was not reached in the grade ≥ 2 irAE group versus 24 months in the grade 0–1 irAE group (hazard ratio [HR] 1.81; 95% confidence interval [CI] 1.04–3.17; *p *= 0.036; Fig. 1A). Similarly, median progression-free survival (PFS) was prolonged in patients with grade ≥ 2 irAE (13 vs. 6.1 months; HR 1.65; 95% CI 1.04–2.63; *p* = 0.032; Fig. 1B). We confirmed this observation in two independent cohorts of 338 and 147 NSCLC patients treated with single-agent anti-PD-1 or combined with chemotherapy in Canada and Japan respectively (Table S2–S3). In both NSCLC cohorts, patients who developed grade ≥ 2 irAE experienced prolonged OS of 29.9 versus 15.4 months (HR 1.73; 95% CI 1.29–2.33; p = 0.001) in the Canadian cohort (Fig. 1C) and 41.5 vs. 15.3 months (HR 1.91; 95% CI 1.49–2.47; *p* = 0.007) in the Japanese cohort (Supplementary Fig. S1B). In addition, PFS was significantly prolonged in both cohorts of patients who developed grade ≥ 2 irAE (Supplementary Fig. S1A, S1C). After adjusting for immortal time bias, the association between grade ≥ 2 irAE and prolonged OS remained significant in both NSCLC cohorts, even though we observed a reduction in the hazard ratio (Canadian HR = 1.73 vs 1.65; Japanese HR = 1.91 vs 1.86). In the melanoma cohort, significance was not reached, but a numerical difference was observed (HR = 1.81 vs 1.36) (Supplementary Fig. S1D).

To investigate potential microbiome correlates, we performed shotgun metagenomics sequencing on available fecal samples from 45 melanoma patients collected prior to ICI initiation. We observed a decrease in alpha diversity in patients with grade ≥ 2 irAE as reflected by the Shannon index (*p* = 0.053) and showed a distinct microbiome composition represented by beta diversity (*p* = 0.032) (Fig. 1D, E). Linear Discriminant Analysis Effect Size (LEfSe) identified, in patients who developed grade ≥ 2 irAE, an enrichment of *Ruminococcus gnavus* (recently reclassified as *Mediterraneibacter gnavus*), *Pseudoruminococcus massiliensis*, and *Streptococcus vestibularis*, bacteria previously associated with inflammatory bowel diseases (Fig. 1F) [32, 33]*.* In contrast, patients in the grade 0–1 irAE group had higher abundances of bacteria associated with gut homeostasis, including *Ruminococcus bicirculans, Bifidobacterium bifidum*, and* Roseburia* sp. AF0212 [34–36]*.* Similarly, in an independent cohort of Canadian patients with NSCLC, we observed a significant reduction in alpha diversity (Shannon index, *p* = 0.028) but no significant differences in beta diversity. Moreover, no taxa overlapped with grade ≥ 2 irAE-associated signature from the melanoma cohort (Supplementary Fig. S1E–S1G).

We next focused on the subset of melanoma patients who developed ir-colitis, as it represents one of the most common irAE. Among the 45 melanoma patient samples profiled, 14 developed ir-colitis (any grade). No differences were observed in alpha or beta diversities between these groups (Supplementary Fig. S1H–S1I). However, taxonomic differences revealed that *Lachnospiraceae* spp. were enriched in ir-colitis, whereas the two probiotics *Bifidobacterium dentium* and *Bifidobacterium pseudocatenulatum* were enriched among the patients without ir-colitis [37, 38] (Supplementary Fig. S1J).

To assess whether baseline microbiome composition was associated with clinical benefit, we stratified melanoma patients based on PFS, as has been done in previous studies [10, 11]. Patients with PFS above the cohort median (16.9 months) referred to as responders (R) exhibited significantly higher alpha diversity and distinct microbiome clustering by beta diversity (Fig. 1G, H). LEfSe analysis identified *Ruminococcus bicirculans*,* Ruminococcus lactaris*, and *Faecalibacterium* SGB15315 as enriched in this favorable-outcome group (Fig. 1I).

To further disentangle microbial shifts associated with response and irAE, we performed multivariable modeling using MaAsLin2. This analysis revealed condition-specific microbial signatures, interpreted relative to the irAE/ICI responder group, which served as the reference. In the melanoma cohort, we observed relative enrichment of *Streptococcus vestibularis* in patients with both irAE/ICI response, consistent with LEfSe results, whereas *Parabacteroides merdae* was relatively decreased in the same group (Fig. 1J). In the NSCLC cohort, only an uncultivated *Proteobacteria* spp. was relatively enriched in the irAE/ICI response group (Supplementary Fig. S1K). Finally, to increase statistical power in this multivariable model, we pooled the melanoma and NSCLC cohorts and found *Holdemanella porci* as enriched in irAE/ICI responder patients (Supplementary Fig. S1L). These findings suggest that a distinct gut microbiome composition is associated with the development of both irAE and response.

### Culturomics identifies a distinct microbial profile in patients who developed ir-colitis

To complement metagenomic analysis and enable the isolation of viable bacterial species, we performed culturomics on stool samples from patients with advanced melanoma (*n* = 18) and NSCLC (*n* = 14) treated with ICI. This high-throughput culture-based method relies on prolonged incubation in optimized media under diverse atmospheric conditions, followed by matrix-assisted laser desorption/ionization-time-of-flight (MALDI-TOF) mass spectrometry identification, allowing the recovery of a broad range of taxa, including low-abundance and previously uncultured [30, 31].

**Fig. 2:**
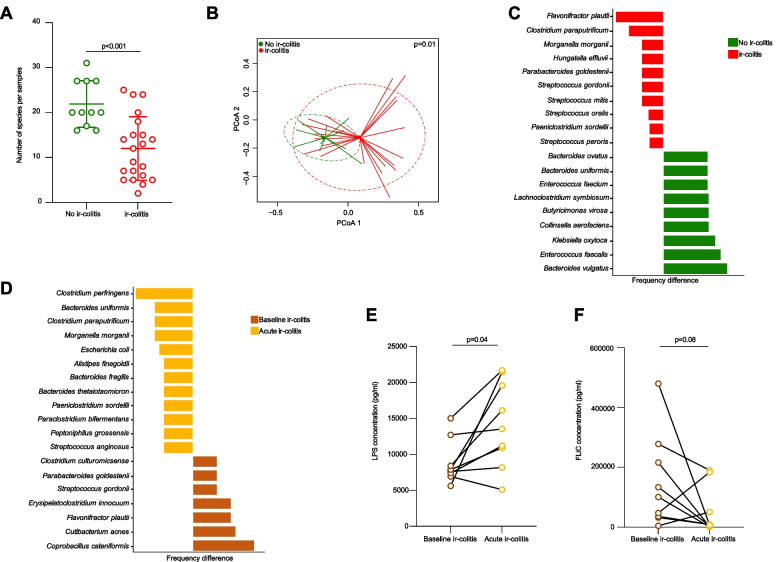
Culturomics of patients who developed ir-colitis identifies a distinct microbial profile associated with inflammatory dysbiosis. **A** Dot plot representation of the number of unique bacterial species (species richness) isolated from fecal samples of patients with advanced melanoma (*n* = 18) and non-small cell lung cancer (NSCLC, *n* = 14) stratified according to the occurrence of immune-related colitis (ir-colitis, *n* = 21) or its absence (no ir-colitis, *n* = 11). Data are presented as mean ± standard error of the mean (SEM). Statistical comparison between groups was performed using an unpaired, non-parametric Mann–Whitney test. **B** Beta diversity was assessed using presence/absence-based Jaccard distance and visualized with principal coordinates analysis (PCoA). Each point represents a sample, and ellipses indicate the 95% confidence interval of group centroids. Group differences were tested using permutational multivariate analysis of variance (PERMANOVA). **C**, **D** Bar plot representation of bacterial taxa showing significant differences in prevalence based on presence/absence between baseline ir-colitis and acute ir-colitis groups. The length of the bars indicates the magnitude of the difference in prevalence. Dot plot showing fecal **E** lipopolysaccharide (LPS) and **F** flagellin (FLiC) concentration between paired patient samples (*n* = 9) at baseline and at the time of appearance of colitis (acute ir-colitis). Statistical comparison between groups was performed using a paired, non-parametric Wilcoxon test

Among the 32 patients analyzed, 21 (66%) developed ir-colitis, and 11 (34%) did not. In the ir-colitis group, fecal samples were collected either at baseline before ICI initiation (*n* = 9) or at the time of acute ir-colitis (*n *= 12), with some patients contributing samples at both time points. Culturomics revealed a significant reduction in the absolute number of isolated bacteria in the ir-colitis samples (mean = 12 species), comprising pooled baseline and acute ir-colitis samples, compared to the no-ir-colitis group (mean = 22 species; *p* < 0.001), with a marked reduction in anaerobic species (Fig. 2A; Supplementary Fig. S2A). Analysis of beta diversity revealed a significant difference in overall composition between the two groups (*p* = 0.01) (Fig. 2B). We observed that four different *Streptococcus* species, as well as *Clostridium perfringens* and *Paeniclostridium sordellii*, were more frequently detected in the ir-colitis group (Fig. 2C), consistent with bacteria previously associated with inflammatory bowel disease and irAE [39, 40]. Conversely, beneficial taxa of the phylum *Bacteroidota*, as well as *Lachnoclostridium symbiosum* and *Butyricicoccus virosa*, known producers of short-chain fatty acids (SCFA) [41], were more frequently isolated in patients who did not develop ir-colitis.

We next compared microbial profiles within the ir-colitis group at two time points: baseline (*n* = 9) and at the onset of colitis (acute ir-colitis, *n* = 12). Neither species richness nor beta diversity differed significantly between these subgroups (Supplementary Fig. S2B–S2C). However, acute ir-colitis samples were enriched in taxa previously associated with inflammation, such as *Paraclostridium bifermentans, Paeniclostridium sordellii*, and *Escherichia coli* [42–44]. While baseline samples harbored a higher prevalence of *Erysipelotrichoclostridium innocuum, Flavonifractor plautii*, and *Streptococcus gordonii* (Fig. 2D).

We then stratified patients according to the median PFS, as performed in the metagenomics analysis (Fig. 1J). *Bacteroides fragilis* was more frequently detected in responders but absent from the baseline versus acute ir-colitis comparison. *Escherichia coli*, enriched at the time of acute ir-colitis, also tended to be more prevalent in responders. In contrast, non-responders showed a higher prevalence of *Anaerocolidibacter massiliensis*, which was not detected in acute ir-colitis samples, as well as *Enterocloster bolteae* and *Enterococcus casseliflavus*, both previously associated with an unfavorable response to ICI [45–47] (Supplementary Fig. S2D).

To assess whether microbial differences between baseline and acute ir-colitis contributed to more inflammation, we analyzed paired samples using HEK-Blue™ hTLR4 cells, a human embryonic kidney (HEK) cell line engineered to express human Toll-like receptor 4 (TLR4), along with its adapter proteins myeloid differentiation factor 2 (MD-2) and cluster of differentiation 14 (CD14). These cells can secrete the embryonic alkaline phosphatase (SEAP) in response to lipopolysaccharide (LPS)-induced activation of the nuclear factor kappa B (NF-κB) pathway [48]. Exposure to fecal supernatant from acute ir-colitis samples triggered significantly higher TLR4 activation compared to baseline samples, suggesting increased LPS levels at the time of ir-colitis (Fig. 2E). In contrast, HEK-Blue™ hTLR5 cells, which respond to flagellin (FLiC), showed a reduced NF-κB response upon exposure to acute samples, reflecting lower FLiC concentrations during active ir-colitis (Fig. 2F).

Together, these findings reveal that patients who develop ir-colitis harbor reduced microbial diversity and are enriched for pro-inflammatory bacteria. Upon the onset of ir-colitis, fecal samples exhibit enhanced LPS-driven signaling and a shift toward a more inflammatory microbial composition. These results underscore the dynamic nature of the gut microbiome in the pathogenesis of irAE in ICI-treated cancer patients.

### Transfer of fecal microbiota from a patient with ir-colitis to mice induces intestinal inflammation and enhances ICI anti-tumor responses

**Fig. 3:**
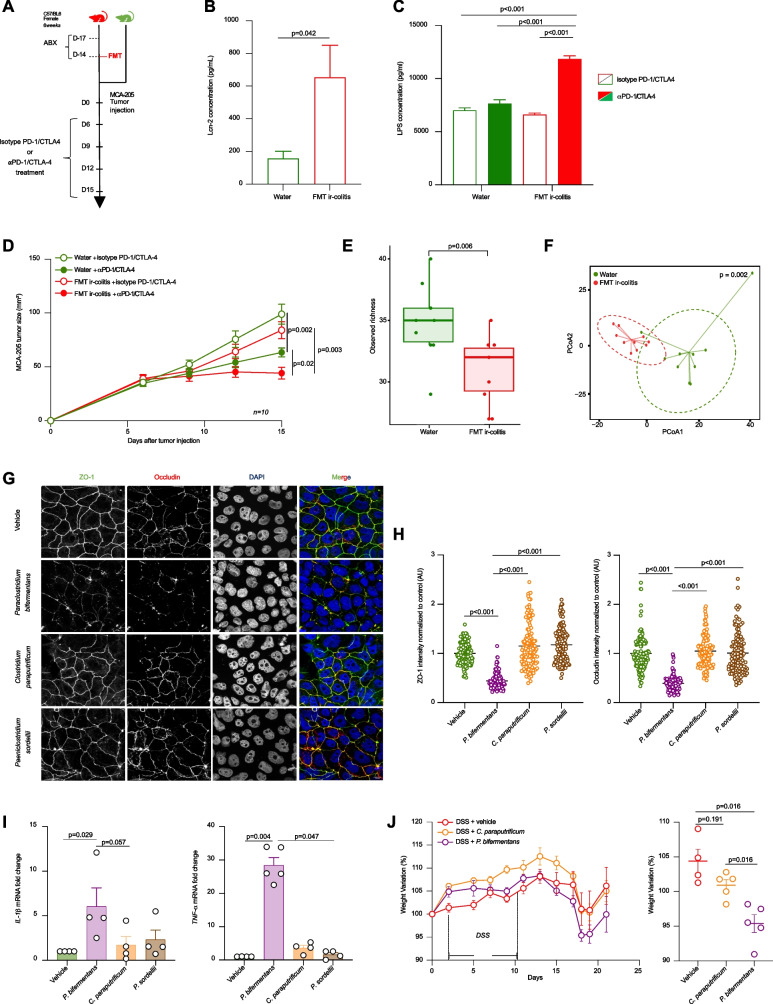
Transfer of fecal microbiota from an ir-colitis patient to mice induces intestinal inflammation and enhances ICI anti-tumor responses. **A** Schematic representation of the fecal microbiota transfer (FMT) experimental design. Antibiotic-treated mice were colonized with a fecal sample derived from a patient who developed immune-related colitis (FMT ir-colitis) or received water (water) during ICI treatment. Mice were subsequently injected with MCA-205 tumor cells and treated with immune checkpoint inhibitors (anti–CTLA-4 + anti–PD-1) or an isotype control. **B** Comparison of the fecal lipocalin-2 (Lcn-2) concentration (pg/ml) between the water control (*n* = 4) and FMT-treated mice (*n* = 8). The FMT group includes mice transplanted with fecal material from two distinct patients, used across two independent murine FMT experiments. Data are presented as mean ± standard error of the mean (SEM). Statistical comparison between groups was performed using an unpaired, non-parametric Mann–Whitney test. **C** Grouped bar plot showing the concentration of fecal lipopolysaccharides (LPS) in water-treated (green) and FMT-treated (red) mice. Each group is further subdivided according to immune checkpoint inhibitors (ICI) treatment: solid bars represent mice treated with anti-PD-1/CTLA-4 antibodies, while empty bars represent mice treated with isotype control. Bars indicate mean ± standard error of the mean (SEM). Statistical comparison between groups (*n* = 4 for each group) was performed using ordinary one-way ANOVA followed by Tukey’s multiple comparisons test. **D** Tumor growth kinetics of subcutaneous MCA-205 implanted in water control (green) and FMT-treated (red) mice in the presence of anti-PD-1/antiCTLA-4 treatment or isotype control. Data are pooled from two independent experiments (*n* = 10 mice per group) and presented as mean ± standard error of the mean (SEM). Statistical comparisons were performed on endpoint tumor size at the time of sacrifice using an unpaired, non-parametric Mann–Whitney test. **E** Alpha diversity of the water and FMT ir-colitis mice gut microbiome assessed by 16S rRNA gene sequencing, represented by the observed richness index. The box represents the interquartile range, the median line indicates the median, and the whiskers represent the extreme values. **F** Beta diversity assessed using principal coordinates analysis (PCoA) based on Bray-Curtis dissimilarity. Each point represents a fecal sample from a mouse treated with either water (Water group, green) or a fecal microbiota transplant (FMT group, red). The ellipses represent the 95% confidence intervals around each group. **G** Representative immunofluorescence image of Caco-2 differentiated cells treated with *P. bifermentans*, *C. paraputrificum*, *P. sordellii* or vehicle control. Cells were fixed with 4% PFA and stained with Zonula Occludens-1 (ZO-1, green), Occludin (red), and DAPI (blue) to visualize tight junctions and nuclei. Scale bar = 10 μm. Quantification of fluorescence intensity (arbitrary units, AU) of **H** ZO-1 and Occludin across treatment groups. Each dot represents an individual cell (*n* = 30 for each group); *n* = 4 independent experiments. Data are presented as mean ± standard error of the mean (SEM). Statistical comparison between groups was performed using the Kruskal-Wallis test, followed by Dunn’s multiple comparison test. **I** Relative IL-1β and TNF-α expression in Caco-2 cells treated with vehicle (blue), *P. bifermentans* (purple), *C. paraputrificum* (orange), or *P. sordellii* (brown), measured by qPCR. Expression was normalized to *ACTB* and *HPRT1* and shown as fold change vs. control. Symbols represent independent experiments (*n* = 4–5/group). Data = mean ± SEM. Groups compared by Mann–Whitney test. **J** Weight variation (%) in mice treated with *P. bifermentans* or *C. paraputrificum* in the presence of dextran sodium sulfate (DSS), compared with DSS alone. (Left) Kinetics of weight variation before the start of DSS treatment until the end of the experiment day 21. Curves represent the mean ± SEM (*n* = 5 mice/group). (Right) Dot plot representation at day 18 (8 days after the end of DSS treatment). Each dot represents an individual mouse. Data are presented as mean ± standard error of the mean (SEM). Statistical comparisons were performed at the time of sacrifice using an unpaired, non-parametric Mann–Whitney test.

To investigate the contribution of the gut microbiome to the development of ir-colitis and its impact on anti-tumor immunity, we performed FMT in antibiotic-treated specific pathogen-free (SPF) mice using a fecal sample from a cancer patient who developed ir-colitis [47]. Fourteen days post-FMT, mice were subcutaneously inoculated with MCA-205 tumor cells and treated with either anti-PD-1/anti-CTLA-4 or isotype control (Fig. 3A). FMT from the ir-colitis patient did not recapitulate the clinical signs of inflammation, such as weight loss or changes in the colon length (Supplementary Fig. S3A–S3B). However, a significant increase in fecal lipocalin-2 (Lcn-2), a marker associated with intestinal inflammation, was observed compared to the water control group (Fig. 3B). Furthermore, LPS levels were significantly elevated in the feces of FMT-recipient mice treated with anti-PD-1/CTLA-4 compared to the water control group, suggesting enhanced gut inflammation post-FMT (Fig. 3C).

Regarding tumor responses, no differences were noted among isotype-treated groups. However, mice receiving ir-colitis FMT followed by anti-PD-1/CTLA-4 exhibited reduced tumor size compared to the dual-ICI-treated water control mice (Fig. 3D). 16S rRNA gene sequencing of stool samples revealed a significant reduction in alpha diversity, measured by the observed richness index in the FMT group compared to the water group mice (Fig. 3E). These analyses also revealed significant differences in the beta diversity of the gut microbiome between mice receiving ir-colitis FMT compared to water control (Fig. 3F). Random forest classification analysis identified *Enterohabdus* as strongly enriched in the FMT ir-colitis group, while *Romboutsia* and *Eubacterium xylanophilum* were more abundant in the water control group (Supplementary Fig. S3C).

To identify candidate microbes contributing to the observed inflammation in the FMT-treated mice, we reviewed bacteria isolated by culturomics from the patient’s stool at baseline and during acute ir-colitis. Among the bacteria isolated from this patient, *Paraclostridium bifermentans* was detected exclusively at the time of acute ir-colitis and was absent at baseline (Supplementary Fig. S3D). This enrichment pattern was also observed in additional patients with ir-colitis, supporting its association with colitis-associated dysbiosis (Fig. 2D). Given this association, we hypothesized that *P. bifermentans* or related species, *Clostridium paraputrificum* and *Paeniclostridium sordellii*, all members of the *Clostridiaceae* family and detected in the ir-colitis patients, may contribute to inflammation.

To test their impact on epithelial integrity, differentiated human Caco-2 monolayers were co-cultured for 4 h with *P. bifermentans*, *C. paraputrificum*, or *P. sordellii*. Zonula occludens-1 (ZO-1) and Occludin staining intensities were significantly reduced only in the presence of *P. bifermentans*, indicating tight junction disruption (Fig. 3G–H). Interestingly, this effect was only observed with whole bacteria *P. bifermentans*; the cell-free supernatant (CFS) did not impair barrier integrity and even increased ZO-1 (Supplementary Fig. S3E–S3F). Reverse transcription quantitative polymerase chain reaction (RT-qPCR) analysis confirmed that *P. bifermentans* triggered a robust inflammatory response in Caco-2 cells, significantly upregulating interleukin (IL)−1β and TNF-α transcripts. No significant cytokine induction was observed with *C. paraputrificum, P. sordellii*, or the control (Fig. 3I).

To validate these findings in vivo, we administered *P. bifermentans* or *C. paraputrificum* orally to mice in a colitis model. Given the limited microbial diversity of laboratory mice and their reduced susceptibility to immune-mediated diseases, we co-administered dextran sodium sulfate (DSS) to disrupt the epithelial barrier and promote colonic inflammation [49]. Mice orally gavaged with *P. bifermentans* exhibited significantly greater weight loss compared to those receiving *C. paraputrificum* or vehicle, consistent with a more severe colitis phenotype (Fig. 3J).

Collectively, these results identify *P. bifermentans*, isolated during ir-colitis, as a pro-inflammatory bacterium capable of disrupting epithelial barrier integrity and enhancing ICI efficacy. Importantly, these effects appear to require the presence of live bacteria rather than soluble factors in the supernatant, highlighting a direct interaction with the host mucosa.

### Metabolites produced by *P. bifermentans* promote ICI responses

**Fig. 4:**
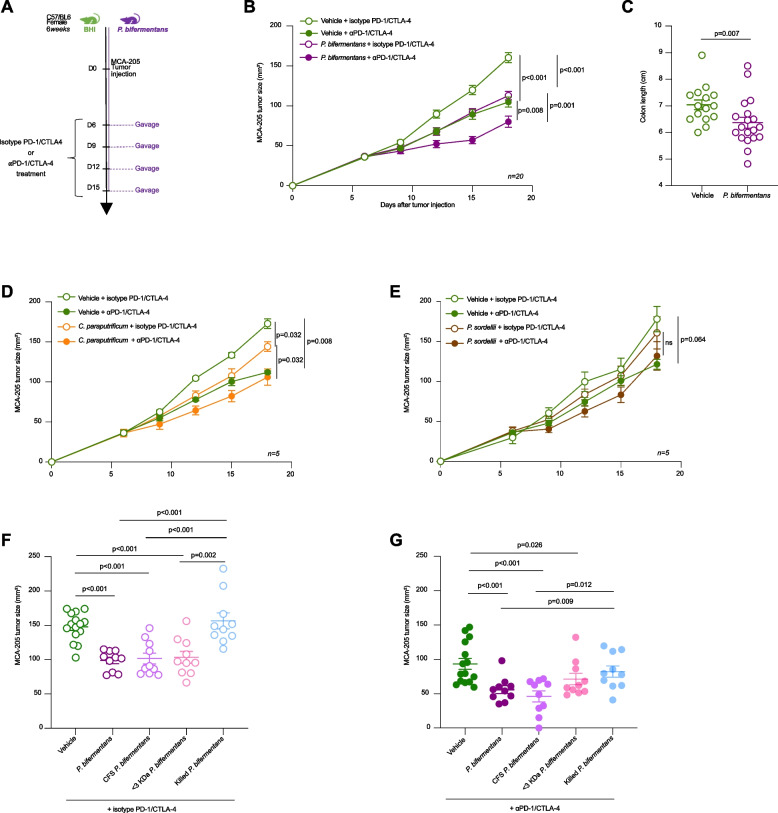
Metabolites produced by *P. bifermentans *promote ICI responses. **A** Schematic representation of the *P. bifermentans* oral gavage experimental design. **B** Tumor growth kinetics of subcutaneous MCA-205 implanted in vehicle (BHI) and *P. bifermentans*-treated mice in the presence of anti-PD-1-CTLA-4 treatment or isotype control. Data are pooled from 4 independent experiments (n = 20 mice for each group) and presented as mean ± standard error of the mean (SEM). Statistical comparisons were performed on endpoint tumor size at the time of sacrifice using an unpaired, non-parametric Mann–Whitney test. **C** Colon length comparison between vehicle (BHI; *n* = 15) and *P. bifermentans* mice (*n* = 19) at the time of sacrifice. Each dot represents an individual mouse. Data are presented as mean ± standard error of the mean (SEM). Statistical comparison between groups was performed using an unpaired, non-parametric Mann–Whitney test. **D** Tumor growth kinetics of subcutaneous MCA-205 and orally supplemented with vehicle (BHI) or *C. paraputrificum* and treated with anti-PD-1/CTLA-4 treatment or isotype control (*n* = 5 mice per group). Data are presented as mean ± standard error of the mean (SEM). Statistical comparisons were performed on endpoint tumor size at the time of sacrifice using an unpaired, non-parametric Mann–Whitney test. **E** Tumor growth kinetics of subcutaneous MCA-205 and orally supplemented with vehicle (BHI, green) or *P. sordellii*. Comparison of the MCA-205 tumor size at the time of sacrifice. Each color corresponds to a distinct experimental group: vehicle (BHI, green), *P. bifermentans* (purple), cell-free supernatant of *P. bifermentans* (CFS, magenta), less than 3 kDa fraction of the cell-free supernatant of P. bifermentans (< 3 kDa, pink) and the heat-killed *P. bifermentans* (HK, blue). **F** Empty plot represents mice treated with isotype control, while **G** solid plot represents mice treated with anti-PD-1/CTLA-4. Each dot represents an individual mouse (*n* = 10–15 for each group). Data are presented as mean ± standard error of the mean (SEM). Statistical comparison between groups was performed using an unpaired, non-parametric Mann–Whitney test

To assess the anti-tumor potential of *P. bifermentans*, *C. paraputrificum*, and *P. sordellii,* mice bearing MCA-205 tumors were orally gavaged every 3 days and treated with either anti-PD-1/CTLA-4 antibodies or an isotype control (Fig. 4A). Compared to the isotype-treated vehicle group, oral gavage of *P. bifermentans* significantly reduced tumor growth. Moreover, the combination of *P. bifermentans* with anti-PD-1/CTLA-4 showed an additive anti-tumor response relative to the vehicle control anti-PD-1/CTLA-4 group (Fig. 4B). In contrast, *C. paraputrificum* and *P. sordellii* bacteria failed to show any additive effect when combined with dual ICI (Fig. 4D, E). In these experiments, colon lengths were shorter only in the *P. bifermentans*-supplemented mice compared to the control group. This difference was not seen in both *C. paraputrificum* and *P. sordellii* (Fig. 4C; Supplementary Fig. S4A–S4B).

To evaluate whether *P. bifermentans*-derived metabolites were sufficient for the observed anti-tumor effect, we next tested the bacterial CFS, its fraction lower than 3 kDa, and the heat-killed bacteria. Unlike our in vitro toxicity experiment, oral administration of the CFS alone suppressed tumor growth compared to isotype controls (Fig. 4F). When combined with anti-PD-1/CTLA-4, the CFS led to an additive anti-tumor effect (Fig. 4G). In addition, the less than 3 kDa filtered fraction of the supernatant retained this activity on natural tumor growth and recapitulated the ICI-potentiating effects of the unfractionated supernatant.

Collectively, these findings demonstrate that the anti-tumor effects of *P. bifermentans* are mediated by a secreted, low-molecular-weight metabolite (< 3 kDa) and do not require live bacterial cells.

## Discussion

In this multicohort study combining metagenomics and culturomics, we confirm that patients developing irAE tend to have improved clinical response, supporting a positive correlation between autoimmune inflammation and anti-tumor activity. Baseline metagenomic analysis from our melanoma cohort showed reduced bacterial diversity and an increase in specific pro-inflammatory species such as *Streptococcus vestibularis*. We found signatures specific concurrently present in both irAE and response states, namely, *Streptococcus vestibularis* and *Holdemanella porci.* Using culturomics at the time of ir-colitis, we identified the enrichment of *Paraclostridium bifermentans.* This bacterium damaged the intestinal barrier and triggered inflammation in vitro and showed an anti-tumor effect in vivo via a metabolite smaller than 3 kDa. These results support the concept that some pro-inflammatory bacteria may simultaneously drive toxicity and enhance ICI efficacy.

Improved survival outcomes in patients developing irAE have been consistently reported in several meta-analyses. In line with our findings, Fan et al. analyzed 9,156 patients and observed significant improvements in OS (HR 0.51; 95% CI 0.41–0.59) and PFS (HR 0.54; 95% CI 0.46–0.62), particularly in melanoma and NSCLC [21]. Liang et al. confirmed these results in NSCLC but noted that the benefit was reduced in grade ≥ 3 irAE, especially gastrointestinal ones [50].

This association remains debated due to immortal-time bias (ITB), whereby patients must survive a certain period to develop irAE, introducing a time-dependent bias if toxicity is analyzed as a fixed variable. Dall’Olio et al. showed that ignoring ITB can overestimate the benefit of irAE, shifting HRs from 0.41 to 0.61 for OS and from 0.47 to 0.66 for PFS [23]. Similarly, Tamura et al. found that landmark analyses and extended Cox models reduced the effect on PFS while preserving a favorable OS association [51]. Similarly, in our three cohorts, correcting for ITB, we observed a decrease in HR. In the two large cohorts of NSCLC, the p-value remained significant despite correcting for this bias. Baseline metagenomics profiling of melanoma patients revealed reduced alpha diversity in those developing irAE, along with a distinct beta diversity composition, consistent with previous observations [15, 16, 52]. We observed enrichment of several *Streptococcus* spp. and *Ruminococcus gnavus* in patients who later developed irAE. In the ir-colitis subgroup, *Lachnospiraceae GGB33746* was enriched. Notably, while low diversity was linked to irAE, patients with prolonged PFS had higher alpha diversity and enrichment in *R. bicirculans*, *R. lactaris*, *B. gluceraseae*, and *Blautia SGB4815*.

Together, these findings highlight an apparent divergence between microbiome features associated with irAE and those associated with improved clinical responses. Indeed, this divergence suggests that response-associated and irAE-associated microbiomes may not be due to a single microbial state, but rather to an overlapping microbiome configuration. In line with these observations, Chaput et al. proposed *Faecalibacterium* spp. as a common predictor of both response and toxicity, and we identified in our cohort *Streptococcus vestibularis* and *Holdemanella porci *in both contexts [53]. However, when attempting to define a group of bacteria associated with irAE and ICI response, only *Holdemanella porci* was consistently increased in this group, suggesting that the overlap between these two microbiome states may be limited. Current evidence suggests that the baseline microbiome may be more predictive of ICI efficacy than of irAE. Compared to irAE, increased baseline diversity and overrepresentation of *Ruminococcus* spp., *Akkermansia muciniphila*, and *Blautia* spp. have been linked to response, while *Enterocloster* spp. and *Hungatella* spp. to resistance [54, 55]. Multiple potential models may explain this apparent disparity. First, efficacy and toxicity may be driven by overlapping but distinct microbiome-immune interactions, where some taxa or microbial components may support anti-tumor immunity, and others favor inflammation leading to toxicity. Second, temporal dynamics after ICI initiation, or during irAE episodes, may be critical to explain differences between response and irAE-associated microbiome. A longitudinal metagenomic study revealed that the gut microbiome of advanced melanoma patients treated with ICI showed dynamic changes after therapy initiation relative to baseline, with distinct microbial trajectories associated with different clinical contexts, including treatment response and irAE development [15]. Additionally, Shang et al. observed a marked and transient shift at the time of ir-colitis, characterized by reduced diversity and inflammatory bowel disease-like configuration, before returning to baseline after resolution [56]. Despite the absence of clear baseline differences in that study, these longitudinal data support the idea that the microbiome associated with irAE may evolve dynamically before, during, and after toxicity onset. Third, irAE may, in some patients, be only a downstream consequence of a potent immune activation rather than a prerequisite for effective anti-tumor immunity. Adding to this complexity, irAE may arise from organ-specific mechanisms and vary depending on ICI regimen, each with distinct microbial and immunological signatures [57]. This raises the possibility that different microbiome-immune interactions are linked to irAE and ICI response. Discrepancies between results may also reflect high inter-study variability. Lee et al. showed that response-associated microbiome signatures are relevant but largely cohort-dependent, with limited reproducibility across studies. The authors also reported that irAE were less strongly associated with the gut microbiome compared to treatment response [8]. Variations in geography, sequencing platforms, irAE types, cancer types, and ICI regimen can all influence detected signatures. McCulloch et al. illustrated this complexity by identifying *Lachnospiraceae* spp. linked to both response and toxicity, while *Streptococcus* spp. were associated only with toxicity [16].

To overcome the limitations of metagenomics, we integrated culturomics, which enables the recovery of a diverse set of live bacteria, including low-abundance bacteria, by using aerobic and anaerobic culture conditions [31]. To our knowledge, our study is one of the first to combine metagenomics and culturomics to characterize the links between the microbiome, immune toxicity, and clinical efficacy of ICI. We have also recently published a study identifying bacterial species detected by both approaches, in which we showed that among the 296 species identified by metagenomics, 61 were also isolated by culturomics, while an additional 93 species were exclusively detected by culturomics from fecal samples of NSCLC patients [29].

In the present study, culturomics of ir-colitis patients revealed reduced diversity at baseline with enrichment of *Clostridium paraputrificum*. During acute ir-colitis, the microbiome shifted toward a pro-inflammatory profile with enrichment of *P. bifermentans* and *Escherichia coli*. Notably, in an FMT study for refractory ir-colitis, *E. coli* blooms have been observed at the time of inflammation, and their elimination post-FMT correlated with reduction of ir-colitis symptoms [28]. Importantly, the impact of ICI on the microbiome composition over time remains largely unexplored. One study suggests that bacterial abundances may vary longitudinally according to clinical outcomes; however, these findings require validation in prospective cohorts, with particular emphasis on microbiome shifts associated with irAE.

In antibiotic-treated mice, FMT from an ir-colitis patient increased subclinical markers of colitis (Lcn-2 and LPS) and enhanced the efficacy of dual ICI therapy. Among the bacterial species identified through culturomics, *P. bifermentans* impaired epithelial barrier integrity and exacerbated DSS-induced weight loss, an effect dependent on live bacteria. Establishing an optimal murine model of ir-colitis remains challenging, largely due to the substantial variability in susceptibility to inflammation. In our study, we were unable to reproduce the additional effect of DSS in combination with dual ICI reported by other groups [18, 58]. This discrepancy likely reflects differences in animal facility conditions, as mice housed in highly sterile environments are less prone to developing colitis. Alternative approaches have been proposed, such as conditional deletion of STAT3 in antigen-presenting cells (CD11c-cre Stat3^fl/fl^ mice), which show aggravated intestinal inflammation upon anti-CTLA-4 treatment [59]. However, such models do not faithfully recapitulate the majority of clinical cases of ir-colitis. A promising solution may lie in the use of more *natural* murine models, such as wild-derived mice, which have been shown to develop intestinal inflammation in ICI studies and may therefore better mimic patient phenotypes [17, 49].

Previous studies have shown that *P. bifermentans* aggravates ir-colitis in mice by increasing pro-inflammatory cytokines and altering epithelial integrity [42]. Pramana et al. further demonstrated its enrichment in an *hnRNPI-*knockout mouse model predisposed to colitis, with production of glycocholate, urocanate, and deoxycholate [60]. By contrast, related species such as *Clostridium paraputrificum* and *Paeniclostridium sordellii* are associated with different pathologies; *P. sordellii* produces lethal toxins responsible for systemic syndromes [61], while *C. paraputrificum* is occasionally linked to colonic necrosis but more often to bacteremia and septic arthritis [62, 63]. However, to date, these species have not demonstrated any in vivo anti-tumoral effect, unlike *P. bifermentans* in our mouse model. The anti-tumor effect observed with the latter, including its supernatant fraction of less than 3 kDa, suggests the production of a small molecule with a direct or indirect immunostimulatory action. To the best of our knowledge, this study provides the first functional evidence that a single bacterium is biologically linked to both irAE events and ICI efficacy through distinct mechanisms. One involves epithelial barrier disruption leading to ir-colitis, and the second involves a small molecule that enhances ICI immune responses.

Nevertheless, our study has several limitations. The limited size of some subgroups, notably the baseline/acute ir-colitis matched samples, reduces the scope of longitudinal analyses. Our mouse models cannot fully recapitulate the complexity of patients’ ir-colitis. Finally, we have not yet identified the exact nature of the active metabolite produced by *P. bifermentans*, nor its mechanism of immune action.

At a translational level, our data suggest that some pro-inflammatory bacteria may paradoxically be beneficial to ICI efficacy, which poses a therapeutic challenge. The development of targeted strategies, such as defined bacterial consortia, purified metabolites, or combined approaches, could help to dissociate the beneficial immunological effect from the inflammatory risk.

## Conclusion

We identify *P. bifermentans* as a bacterium with dual potential: promoting ir-colitis and enhancing therapeutic efficacy in patients treated with ICI. These results reinforce the idea that functional analysis of the microbiome, beyond simple taxonomy, is essential to disentangle the paradox between irAE and ICI efficacy.

## Supplementary Information

Supplementary Material 1. Figure S1: Development of irAE is associated with improved clinical outcomes and distinct microbiome signatures.

Supplementary Material 2. Figure S2: Fecal culturomics according to ir-colitis and response status.

Supplementary Material 3. Figure S3: Fecal microbiota transfer from patients with ir-colitis. 

Supplementary Material 4. Figure S4: Colon length from mice after gavage with *C. paraputrificum* or *P. sordellii.*

Supplementary Material 5. Table S1. Baseline clinical characteristics of melanoma patients according to irAE status. Supplementary Table S2. Baseline clinical characteristics of the Canadian NSCLC cohort according to irAE status. Supplementary Table S3. Baseline clinical characteristics of the Japanese NSCLC cohort according to irAE status.

## Data Availability

The sequencing datasets analyzed during the current study are available in the NCBI Sequence Read Archive (SRA) under BioProject accession number PRJNA1314234 (https://www.ncbi.nlm.nih.gov/bioproject/PRJNA1314234). The data are publicly accessible. No custom code or scripts were used in this study. All analyses were performed using standard publicly available software, as detailed in the Methods section.
