# Supplementary figures and images for: Metagenomics and culturomics reveal the dual role of the gut microbiome in the development of immune-related toxicities and the efficacy of immune checkpoint inhibitors in cancer
Source: Microbiome. 2026 May 4;14:170. doi: 10.1186/s40168-026-02419-4 (PMC13288583; doi:10.1186/s40168-026-02419-4)

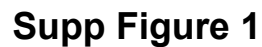

Supplement: Supplementary file 2 — Supplementary Material 1. Figure S1: Development of irAE is associated with improved clinical outcomes and distinct microbiome signatures. [file 40168_2026_2419_MOESM1_ESM.pdf]

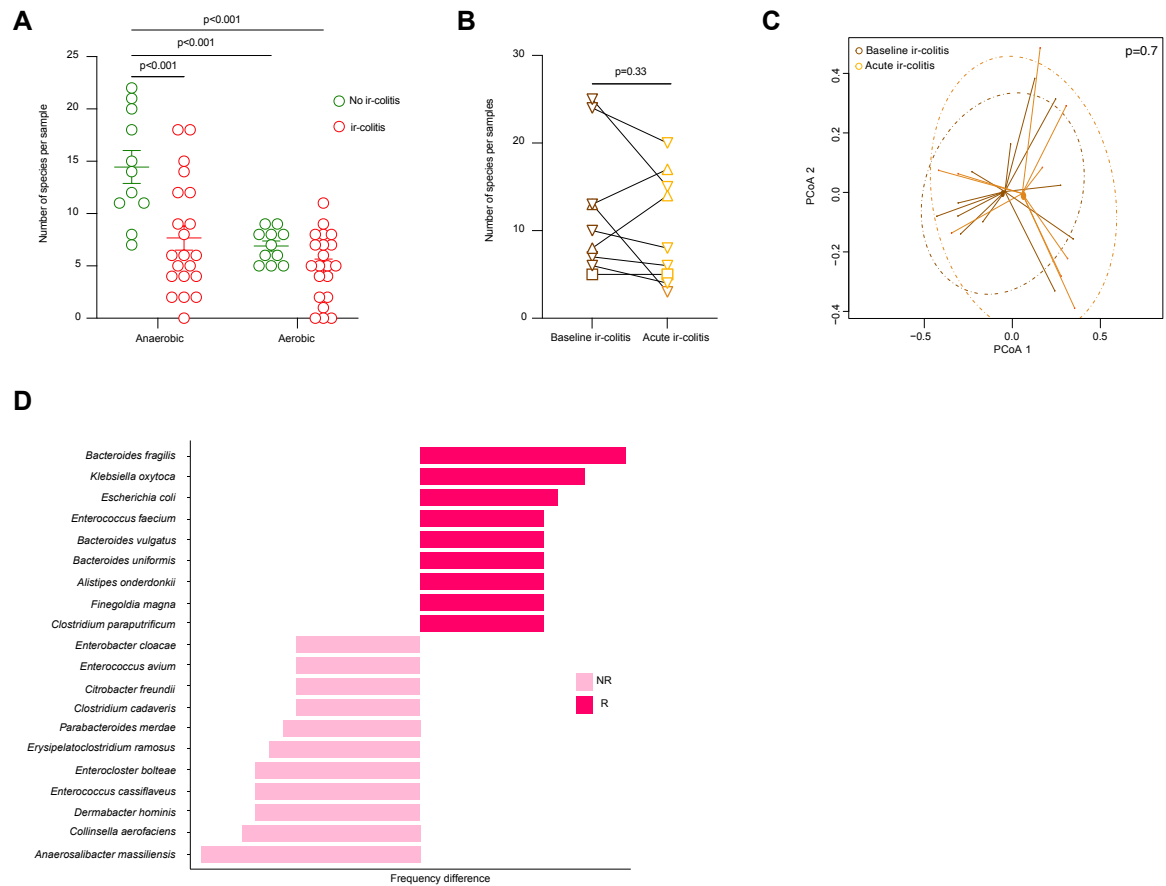

Supplement: Supplementary file 3 — Supplementary Material 2. Figure S2: Fecal culturomics according to ir-colitis and response status. [file 40168_2026_2419_MOESM2_ESM.pdf]

**A**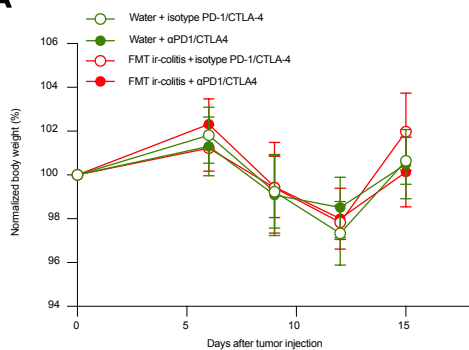**B**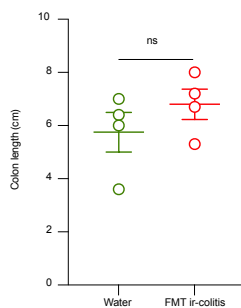**C**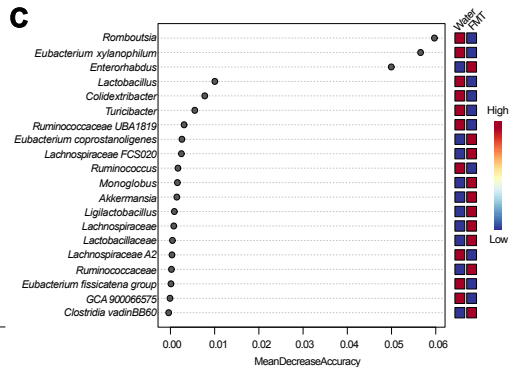**D**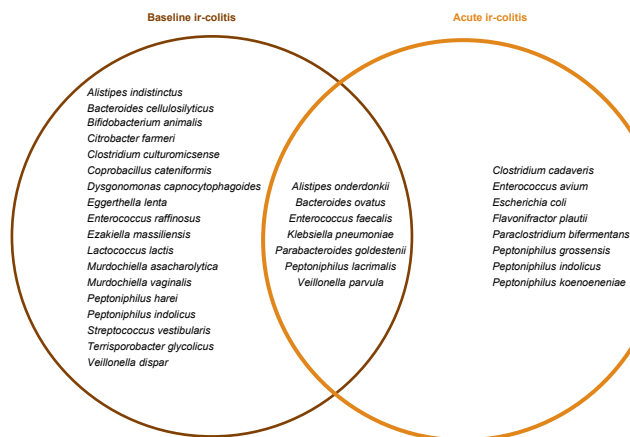**E**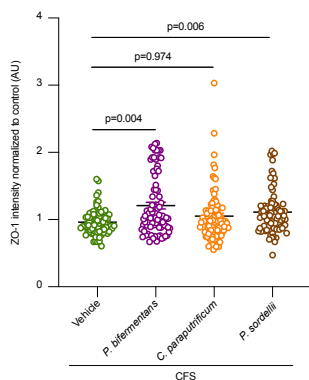**F**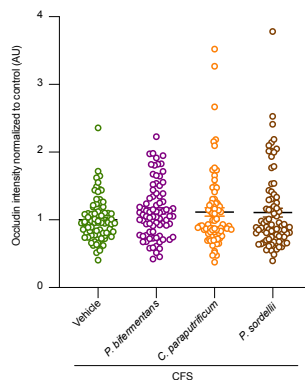

Supplement: Supplementary file 4 — Supplementary Material 3. Figure S3: Fecal microbiota transfer from patients with ir-colitis. [file 40168_2026_2419_MOESM3_ESM.pdf]

**A**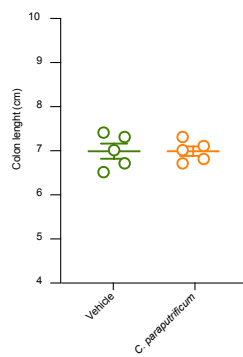**B**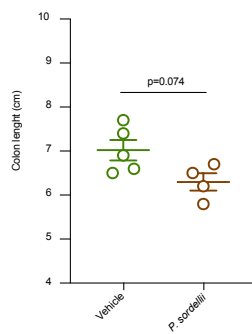

Supplement: Supplementary file 5 — Supplementary Material 4. Figure S4: Colon length from mice after gavage with C. paraputrificum or P. sordellii. [file 40168_2026_2419_MOESM4_ESM.pdf]
