# Supplementary material for: Metagenomics and culturomics reveal the dual role of the gut microbiome in the development of immune-related toxicities and the efficacy of immune checkpoint inhibitors in cancer
Source: Microbiome. 2026 May 4;14:170. doi: 10.1186/s40168-026-02419-4 (PMC13288583; doi:10.1186/s40168-026-02419-4)
Supplement: Supplementary file 6 — Supplementary Material 5. Table S1. Baseline clinical characteristics of melanoma patients according to irAE status. Supplementary Table S2. Baseline clinical characteristics of the Canadian NSCLC cohort according to irAE status. Supplementary Table S3. Baseline clinical characteristics of the Japanese NSCLC cohort according to irAE status. [file 40168_2026_2419_MOESM5_ESM.docx]

| Melanoma | G0-G1 N = 55^1^ | G≥2 N = 49^1^ | p-value^2^ |
| --- | --- | --- | --- |
| **Age** | 62 (54, 72) | 65 (51, 73) | *0.5* |
| Unknown | 2 | 3 |  |
| **Sex** |  |  | *0.4* |
| Female | 11 (20%) | 13 (27%) |  |
| Male | 44 (80%) | 36 (73%) |  |
| **BRAF status** |  |  | *0.3* |
| Negative | 36 (68%) | 27 (57%) |  |
| V600E/K | 17 (32%) | 19 (40%) |  |
| Non-V600E/K | 0 (0%) | 1 (2.1%) |  |
| Unknown | 2 | 2 |  |
| **Stage** |  |  |  |
| IV | 55 (100%) | 49 (100%) |  |
| **Treatment** |  |  | *0.13* |
| Pembrolizumab | 25 (45.5%) | 20 (40.8%) |  |
| Nivolumab | 19 (34.5%) | 11 (22.4%) |  |
| Ipilimumab + Nivolumab | 11 (20%) | 18 (36.7%) |  |
| **Liver metastases at baseline** | 11 (20%) | 14 (29%) | *0.3* |
| Unknown | 1 | 1 |  |
| **Brain metastases at baseline** | 6 (11%) | 14 (29%) | *0.025* |
| Unknown | 1 | 0 |  |
| ^1^ Median (Q1, Q3); n (%) | | | |
| ^2^ Wilcoxon rank sum test; Pearson’s Chi-squared test; Fisher’s exact test | | | |

**Supplementary Table 1. Baseline clinical characteristics of melanoma patients according to irAE status.**

Continuous variables are expressed as median (Q1, Q3), and categorical variables as n (%). Group comparisons were performed using the Wilcoxon rank-sum test for continuous variables, and Pearson’s Chi-squared test or Fisher’s exact test for categorical variables, as appropriate.

*Abbreviations*: irAE: immune-related adverse event, BRAF: B-Raf proto-oncogene, V600E/K: replacement of the valine (V) at amino acid 600 of the BRAF protein by either a glutamate (E) or lysine (K).

| NSCLC (Canadian) | G0-G1 N = 249^1^ | G≥2 N = 89^1^ | p-value^2^ |
| --- | --- | --- | --- |
| **Age** | 68 (62, 72) | 67 (62, 75) | *0.8* |
| **Sex** |  |  | *>0.9* |
| Female | 126 (51%) | 45 (51%) |  |
| Male | 123 (49%) | 44 (49%) |  |
| **PD-L1 status** |  |  | *0.2* |
| <1% | 61 (28%) | 16 (21%) |  |
| >50% | 99 (45%) | 45 (58%) |  |
| 1-49% | 60 (27%) | 17 (22%) |  |
| Unknown | 29 | 11 |  |
| **Stage** |  |  | *0.3* |
| II | 4 (1.6%) | 4 (4.5%) |  |
| III | 40 (16%) | 12 (13%) |  |
| IV | 205 (82%) | 73 (82%) |  |
| **Treatment line** |  |  | *0.2* |
| First | 131 (52.6%) | 37 (41.6%) |  |
| Second or more | 108 (43.4%) | 48 (53.9%) |  |
| Adjuvant | 10 (4%) | 4 (4.5%) |  |
| **Treatment** |  |  | *0.5* |
| Pembrolizumab | 142 (57%) | 49 (55.1%) |  |
| Nivolumab | 81 (32.5%) | 32 (36%) |  |
| Ipilimumab + Nivolumab | 2 (0.8%) | 0 (0%) |  |
| Other | 24 (9.6%) | 8 (8.9%) |  |
| **RECIST** |  |  | *<0.001* |
| PD | 101 (40.6%) | 17 (19.1%) |  |
| SD | 83 (33.3%) | 29 (32.6%) |  |
| PR | 56 (22.5%) | 26 (29.2%) |  |
| CR | 7 (2.8%) | 15 (16.9%) |  |
| NA | 2 (0.8%) | 2 (2.2%) |  |
| ^1^ Median (Q1, Q3); n (%) | | | |
| ^2^ Wilcoxon rank sum test; Pearson’s Chi-squared test; Fisher’s exact test | | | |

**Supplementary Table 2. Baseline clinical characteristics of the Canadian NSCLC cohort according to irAE status.**

Continuous variables are expressed as median (Q1, Q3), and categorical variables as n (%). Group comparisons were performed using the Wilcoxon rank-sum test for continuous variables, and Pearson’s Chi-squared test or Fisher’s exact test for categorical variables, as appropriate.

*Abbreviations*: irAE: immune-related adverse event, PD-L1: Programmed Death-Ligand 1, PD: progressive disease, SD: stable disease, PR: partial response, CR: complete response, NA: not available.

| NSCLC (Japanese) | G0-G1  N = 98^1^ | G≥2  N = 49^1^ | p-value^2^ |
| --- | --- | --- | --- |
| **Age** | 71 (65, 75) | 72 (62, 75) | *0.8* |
| **Sex** |  |  | *0.6* |
| Female | 42 (42.9%) | 19 (38.8%) |  |
| Male | 56 (57.1%) | 30 (61.2%) |  |
| **PD-L1 status** |  |  | *0.5* |
| <1% | 15 (15.3%) | 10 (20.4%) |  |
| >50% | 39 (39.8%) | 19 (38.8%) |  |
| 1-49% | 31 (31.6%) | 17 (34.7%) |  |
| Unknown | 13 (13.2%) | 3 (6.1%) |  |
| **Stage** |  |  | *0.4* |
| III | 6 (6.1%) | 2 (4.1%) |  |
| IV | 68 (69.4%) | 32 (65.3%) |  |
| Recurrence | 24 (24.5%) | 15 (30.6%) |  |
| **Treatment line** |  |  | 0.04 |
| First | 69 (70.4%) | 42 (85.7%) |  |
| Second or more | 29 (29.6%) | 7 (14.3%) |  |
| **Treatment** |  |  | *0.2* |
| Pembrolizumab | 43 (43.4%) | 21 (42.9%) |  |
| Pembro + Chemo | 21 (21.4%) | 21 (42.9%) |  |
| Atezolizumab | 11 (11.2%) | 3 (6.1%) |  |
| Atezo + Chemo | 6 (6.1%) | 1 (2%) |  |
| Nivolumab | 7 (7.1%) | 2 (4.1%) |  |
| Ipilimumab + Nivolumab | 1 (1%) | 1 (2%) |  |
| **RECIST** |  |  | *0.01* |
| PD | 34 (34.7%) | 5 (10.2%) |  |
| SD | 20 (20.4%) | 15 (30.6%) |  |
| PR | 38 (38.8%) | 28 (57.1%) |  |
| CR | 1 (1%) | 0 (0%) |  |
| NA | 5 (5.1%) | 1 (2%) |  |
| ^1^ Median (Q1, Q3); n (%) | | | |
| ^2^ Wilcoxon rank sum test; Pearson’s Chi-squared test; Fisher’s exact test | | | |

**Supplementary Table 3. Baseline clinical characteristics of the Japanese NSCLC cohort according to irAE status.**

Continuous variables are expressed as median (Q1, Q3), and categorical variables as n (%). Group comparisons were performed using the Wilcoxon rank-sum test for continuous variables, and Pearson’s Chi-squared test or Fisher’s exact test for categorical variables, as appropriate.

*Abbreviations*: irAE: immune-related adverse event, PD-L1: Programmed Death-Ligand 1, Chemo: chemotherapy, PD: progressive disease, SD: stable disease, PR: partial response, CR: complete response, NA: not available.
